# Dorsal Visual Pathway Changes in Patients with Comitant Extropia

**DOI:** 10.1371/journal.pone.0010931

**Published:** 2010-06-03

**Authors:** Xiaohe Yan, Xiaoming Lin, Qifeng Wang, Yuanchao Zhang, Yingming Chen, Shaojie Song, Tianzi Jiang

**Affiliations:** 1 State Key Laboratory of Ophthalmology, ZhongShan Ophthalmic Center, Sun Yat-sen University, Guangzhou, People's Republic of China; 2 National Laboratory of Pattern Recognition, Institute of Automation, Chinese Academy of Sciences, Beijing, People's Republic of China; 3 Department of Radiology, First Affiliated Hospital, Sun Yat-sen University, Guangzhou, People's Republic of China; University of Illinois at Chicago, United States of America

## Abstract

**Background:**

Strabismus is a disorder in which the eyes are misaligned. Persistent strabismus can lead to stereopsis impairment. The effect of strabismus on human brain is not unclear. The present study is to investigate whether the brain white structures of comitant exotropia patients are impaired using combined T1-weighted imaging and diffusion tensor imaging (DTI).

**Principal Findings:**

Thirteen patients with comitant strabismus and twelve controls underwent magnetic resonance imaging (MRI) with acquisition of T1-weighted and diffusion tensor images. T1-weighted images were used to analyze the change in volume of white matter using optimized voxel-based morphology (VBM) and diffusion tensor images were used to detect the change in white matter fibers using voxel-based analysis of DTI in comitant extropia patients. VBM analysis showed that in adult strabismus, white matter volumes were smaller in the right middle occipital gyrus, right occipital lobe/cuneus, right supramarginal gyrus, right cingulate gyrus, right frontal lobe/sub-gyral, right inferior temporal gyrus, left parahippocampa gyrus, left cingulate gyrus, left occipital lobe/cuneus, left middle frontal gyrus, left inferior parietal lobule, and left postcentral gyrus, while no brain region with greater white matter volume was found. Voxel-based analysis of DTI showed lower fractional anisotropy (FA) values in the right middle occipital gyrus and right supramarginal gyrus in strabismus patients, while brain region with increased FA value was found in the right inferior frontal gyrus.

**Conclusion:**

By combining VBM and voxel-based analysis of DTI results, the study suggests that the dorsal visual pathway was abnormal or impaired in patients with comitant exotropia.

## Introduction

Comitant strabismus is a common form of strabismus, which affects 1–4.2% of the population [1], [2], [3]. It is characterized by a constant angle of deviation in different directions of gaze. In clinical practice, comitant strabismus also presents with stereopsis impairment, especially in those patients with onset in early childhood, whose stereopsis is substantially impaired. Moreover, stereopsis is still unrestored for many adults with early-onset and long-standing strabismus though they underwent strabismus surgery. These interesting phenomena may suggest that certain regions in the brain, particularly the regions controlling stereopsis, may be affected by early abnormal visual experience.

Previous studies have already suggested that brain plasticity changes can be induced by early abnormal viusal experience, such as strabismus. In primate experiments the number of binocular neurons in the V1 region and the bilateral horizontal connections between V1 areas are reduced by strabismus [4], [5], [6], [7], [8], [9], while those neurons responding to each eye are not affected [4], [5], [6], [7]. Furthermore, optical imaging studies in cat have found that strabismus can induce the segregation of ocular dominance domains in area 17 [10], and modify the connection within area 18 and the connection from area 17 to area 18 [11]. In strabismus cats, the neural deficits are not confined to the visual cortex, but also in the thalamus [12], [13].

Despite growing evidence for brain structure changes in strabismus animals, few studies have focused on the impact of strabismus on human brain. Chan et al. used voxel-based morphometry (VBM) to analyze changes in gray and white matter volume in 10 patients with comitant exotropia compared with 10 healthy volunteers [14]. They found that the gray matter of the occipital eye field (OEF) and parietal eye field (PEF) was of smaller volumes in the strabismus group, while the gray matter of the front eye field (FEF), supplementary eye field (SEF), prefrontal cortex (PFC) and some subcortical areas showed greater volumes in this group. Chan and colleagues proposed that the brain of strabismus patients had plastic changes, such that the oculomotor regions had increased in volume in compensation for the atrophy of visual cortex [14]. However, their study did not account for the potential confounding effect of amblyopia, which is a developmental problem in human brain and also accompanies with stereopsis impairment [15]; in their study, most patients with strabismus have amblyopia and some patients show very small deviation angles. In addition, the magnetic field intensity in the study was as low as 1T, in which spatial resolution was not as high as that in high-field MR system because of lower signal/nosie ratio. Therefore, the effect of comitant strabismus on human brain remains undetermined.

T1-weighted imaging and diffusion tensor imaging (DTI) are widely used in brain research, which are both noninvasive methods to measure the brain structure in neurological disorders. The former technique can be used to detect change in volume of gray and white matter using VBM, which is based on voxel-by-voxel analysis [16]. The latter method can detect the integrity of white matter fibers connectivity via analyzing the abnormality of fractional anisotropy (FA), which is the main indicator reflecting directionality of water diffusion in voxel-based analysis of DTI [17].

Here we used T1-weighted imaging and DTI to explore the structure changes of brain plasticity in comitant strabismus patients with normal corrected visual acuity. Our study aims to investigate the changes in white matter structure of the strabismus patients using optimized VBM and voxel-based analysis of DTI, which can help to elucidate the effect of early abnormal experience on the plasticity of human brain.

## Methods

### Subjects

The study was approved by the Ethics Committee of ZhangShan Ophthalmic Center, Sun Yat-sen University and followed the tenets of the Declaration of Helsinki. All the participants enrolled in the study signed informed consents and received detailed eye examinations, including visual acuity, ocular pressure, refraction, anterior segment anatomy, ophthalmoscopy, binocular alignment, ocular motility, random-dot butterfly stereogram and synoptophore. A total of 13 patients (6 female and 7 male; average age 22.0±2.89 years) with comitant exotropia and 12 normal volunteers (8 female and 4 male; average age 23.17±2.52 years) were enrolled in the study. The mean age at strabismus diagnosis was 5.5±6.6 (range birth to 15 years) and the mean distance exodeviation was 79.7±30.7 prism dioptres (PD) (range 30 to 140). All patients had no stereopsis and the normal subjects had good stereopsis as detected by random-dot butterfly stereogram. The normal volunteers had no history of strabismus. All the subjects had normal corrected visual acuity for both eyes, right-handed, had no intermitent exotropia, other ocular disease or surgery, neurological disorders, or brain abnormality based on MRI scan. Detailed clinical data are shown in Table 1.

**Table 1:** Clinical Characteristics.

	Normal (12)	Extropia (13)	*P* value
Age(Year)	23.2±2.5	22.0±2.9	0.29^a^
Gender	4M/8F	7M/6F	0.30^b^
Age of Onset (Year)		5.5±6.6	
Near Prism Exodeviation (PD)		63.3±32.0	
Distance Prism Exodeviation (PD)		79.7±30.7	
Stereopis (Seconds of arc)	40	none	

a Two sample t-test.

b Pearson Chi-square test.

### Data acquisition

A Siemens Trio 3.0 T MR scanner was used to acquire T1-weighted images and DTI images. The 3D magnetization-prepared rapid gradient-echo imaging (MP-RAGE) sequence was used for structural T1-weighted imaging in a sagital orientation and spin-echo version of echo planar imaging (SE-EPI) sequence for DTI imaging. Parameters were as follows:

Structural imaging: repetition time = 2000 ms; echo time = 2.6 ms; flip angle = 9°; acquisition matrix = 256×256; voxel size = 1×1×1 mm^3^; field of view = 256×224 mm^2^; bandwidth = 200 Hz/PX. The scanning time was about 5 min and a total of 192 images were obtained.

Diffuse tensor imaging: repetition time = 6300 ms; echo time = 82 ms; acquisition matrix = 128×128; field of view = 256×256 mm^2^; voxel size = 2×2×3 mm^3^; axial slices = 45; slice thickness = 3 mm; bandwidth = 1502 Hz/PX. The diffusion sensitizing gradients were applied along 12 nonlinear directions (b = 1000 s/mm^2^), together with an acquisition without diffusion weighting (b = 0 s/mm^2^), average time = 3. The scanning time was about 6 min and a total of 39 images (13×3) were obtained.

### Data processing

The T1-weighted images were analyzed with optimized VBM developed by Good et al [18]. Theses structural images were processed with voxel-based morphometry toolbox (VBM5.1) toolbox (http://dbm.neuro.uni-jena.de/vbm) implemented in the Statistic Parametric Mapping 5 software package (SPM5, Wellcome Department of Cognitive Neurology, London, UK, http://www.fil.ion.ucl.ac.uk/spm), which included normalization, segmentation, modulation and smooth steps. The algorithm from SPM5 and the extension of Hidden Markov Random Field approach were used in the toolbox. The segmented white matter images were modulated with Jacobian determinant to compensate for volume changes during spatial normalization. Then the modulated white matter images are smoothed with an 8 mm full width at half maximization (FWHM) isotropic Gaussian kernel to increase signal-to-noise ratio and improve the ability to detect morphometric variations.

Diffuse tensor images were analyzed with voxel-based analysis of DTI [19], and completed by Statistic Parametric Mapping 2 (SPM2, Wellcome Department of Cognitive Neurology, London, UK) and FSL (FSL, Version 3.3; www.fmrib.ox.ac.uk/fsl) software. The main steps were as follows: 1. Eddy current correction was performed with FMRIB's diffusion toolbox in FSL software for the diffusion weighted images, which were then transformed to corresponding b = 0 images through affine registration. 2. Tensor reconstruction was performed based on DTI, and tensor matrix was diagonalized to obtain eigenvalues λ_1_, λ_2_ and λ_3_, as well as corresponding eigenvectors, then the FA value of each voxel was calculated according to the following formula.
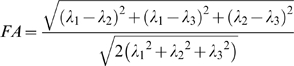
3. b = 0 image of each subject was normalized to the Montreal Neurological Institute (MNI) standard space using EPI template with SPM2. Similarly, the relevant FA images were normalized to MNI space, and their transformation parameters remained the same as b0 images. As a result, the size of each voxel was 2×2×2 mm^3^. 4. Space smoothing was conducted for each FA image with 8 mm FWHM Gaussian kernel.

### Statistical analysis

In the VBM method, two-sample *t*-tests were used to compare the volume of each voxel between strabismus and normal control groups using analysis of covariance, with age and sex as covariate to control the effect of age and sex. The statistical difference was defined when an uncorrected *P*<0.001 was obtained and the cluster size was >20 voxels.

In the VBA method, two-sample *t*-tests were used to compare the FA values between strabismus and normal control groups in voxel-based manner using analysis of covariance, with age and sex as covariate to control the effect of age and sex. The statistical difference was defined when an uncorrected *P*<0.001 was obtained and the cluster size was >30 voxels.

## Results

### Results of Optimized VBM analysis

#### Changes in white matter volumes

**Figure 1:**
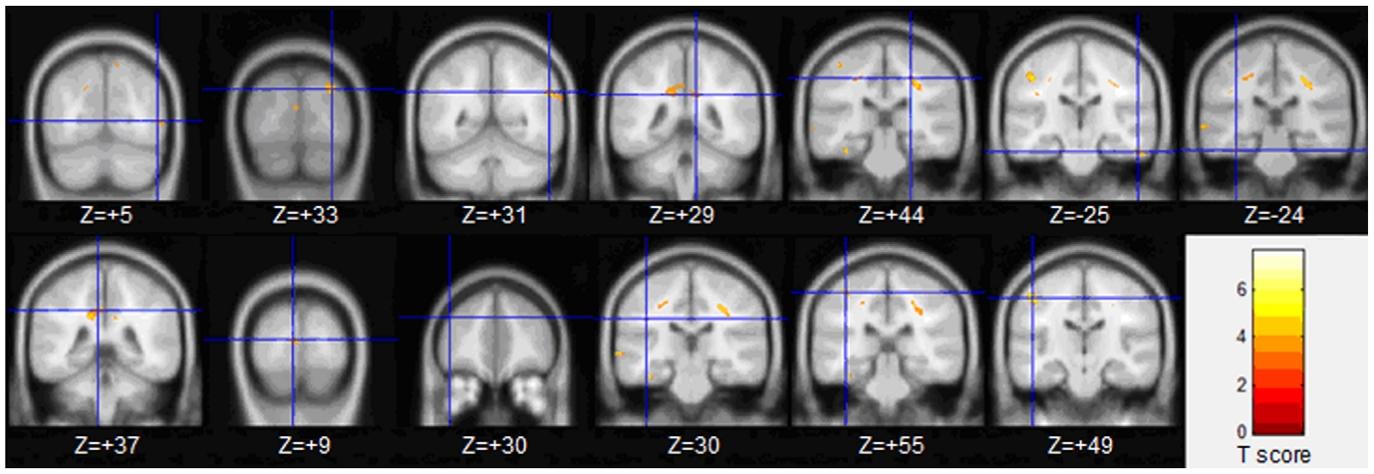
White matter regions with reduced volumes in adult strabismus. Strabismus patients showed smaller white matter in several regions, including the right middle occipital gyrus (Z = +5), right occipital lobe/cuneus (Z = +33), right supramarginal gyrus (Z = +31), right cingulate gyrus (Z = +29), right frontal lobe/sub-gyral (Z = +44), right inferior temporal gyrus (Z = −25), left parahippocampa gyrus (Z = −24), left cingulate gyrus (Z = +37), left occipital lobe/cuneus (Z = +9), left middle frontal gyrus (Z = +30), left inferior parietal lobule (Z = 30), and left postcentral gyrus (Z = +55, Z = +49).

Compared to the normal control group, the strabismus group showed smaller white matter volumes at the right middle occipital gyrus, right occipital lobe/cuneus, right supramarginal gyrus, right cingulate gyrus, right frontal lobe/sub-gyral, right inferior temporal gyrus, left parahippocampa gyrus, left cingulate gyrus, left occipital lobe/cuneus, left middle frontal gyrus, left inferior parietal lobule, and left postcentral gyrus (*P*<0.001, uncorrected; see Table 2 and Figure 1), while no brain region with greater white matter volume was found at the right inferior temporal gyrus.

**Table 2:** Changes of white matter volumes in adult strabismus.

Brain regions	Brodmann	Cluster	T	MNI coordinate		
	area	size	score	x	y	z (mm)
Strabismus<Normal Control						
Right middle occipital gyrus	19, 37	28	4.06	49	−71	5
Right occipital lobe/cuneus	-	243	5.47	31	−83	33
Right supramarginal gyrus	40	153	4.83	54	−52	31
Right Cingulate Gyrus	31	151	4.10	−41	−43	29
Right frontal lobe/sub-gyral	-	561	4.91	25	−29	44
Right inferior temporal gyrus	20	153	5.39	54	−23	−25
Left parahippocampa gyrus	-	28	4.72	−36	−28	−24
Left cingulate gyrus	31	480	4.64	−6	−40	37
Left occipital lobe/cuneus	-	72	4.44	−43	−89	9
Left middle frontal gyrus	46	23	4.18	10	42	30
Left inferior parietal lobule	-	44	4.08	49	26	30
Left postcentral gyrus	3	95	5.33	−325	−31	55
Left postcentral gyrus	1, 2, 4	155	7.64	−47	−20	49

White matter volumes were smaller in strabismus group than in the normal group, at the right middle occipital gyrus, right occipital lobe/cuneus, right supramarginal gyrus, right cingulate gyrus, right frontal lobe/sub-gyral, right inferior temporal gyrus, left parahippocampa gyrus, left cingulate gyrus, left occipital lobe/cuneus, left middle frontal gyrus, left inferior parietal lobule, and left postcentral gyrus. No brain region with greater white matter volume was found.

### Results of voxel-based analysis of DTI

**Figure 2:**
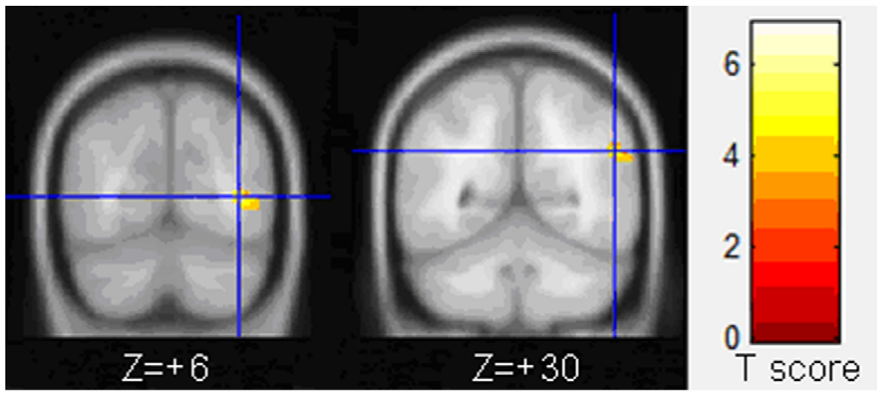
Whiter matter regions with lower FA in adult strabismus. White matter regions with lower FA in strabismus patients, including right middle occipital gyrus (Z = +6) and right supramarginal gyrus (Z = +30).

**Figure 3:**
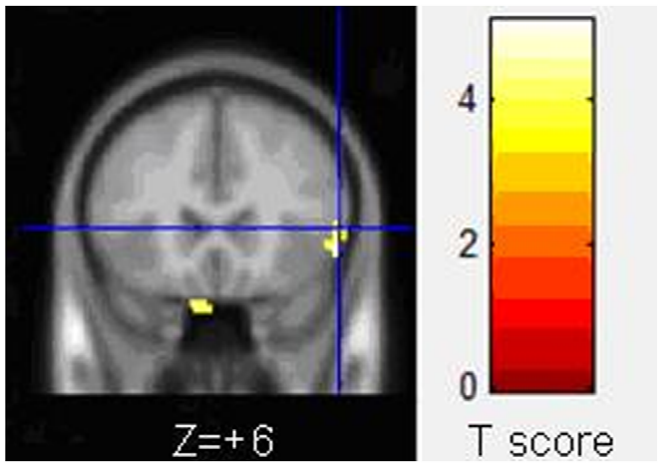
White matter region with increased FA in adult strabismus. White matter region with higher FA in strabismus patients was found in the right inferior frontal gyrus (Z = +6).

Compared to the normal control group, the FA values were decreased in all the right hemisphere of the strabismus group, including middle occipital gyrus and supramarginal gyrus (*P*<0.001, uncorrected; see Table 3 and Figure 2). Right inferior frontal gyrus with increased FA was found in the strabismus group (*P*<0.001, uncorrected; see Table 3 and Figure 3).

**Table 3:** Locations of white matter regions with FA changes in adult strabismus.

Brain regions	Brodmann	Cluster	T	MNI coordinate		
	area	size	score	x	y	z (mm)
Strabismus<Normal Control						
Right middle occipital gyrus	19, 37	93	6.93	38	−70	6
Right supramarginal gyrus	40	77	5.00	52	−54	30
Left cingulate gyrus	31	26	4.05	−4	−36	38
Strabismus>Normal Control						
Right inferior frontal gyrus	47	35	−3.54	58	22	6

There are several white matter regions with significantly lower FA in adult strabismus than that in normal controls by voxel-based analysis of DTI. These regions include the right middle occipital gyrus and right supramarginal gyrus. Region with increased FA in strabismus was found in the right inferior frontal gyrus.

## Disscussion

In our study, VBM and voxel-based analysis of DTI showed that there were structural abnormalities in occipital and parietal areas, especially in the middle occipital gyrus and inferior parietal lobule (supramarginal gyrus), which play important roles in the dorsal visual pathway. Thus the major finding of the study is that the dorsal visual pathway was abnormal in patients with comitant exotropia. The dorsal visual pathway originates from V1 area, passes through V2 and MT areas, and arrives at the inferior parietal lobule. This pathway primarily takes part in the processing of spatial position information and eye movement [20], [21]. But we don't know whether the abnormalities in dorsal visual pathway is the cause or result of the strabismus.

Our VBM results were partially consistent with the previous report by Chan et al, that is, the occipital and parietal lobules showed smaller volumes in strabismus patients. However, there were some conflicting results, with our study showing that no regions with greater volumes were found in strabismus patients, which may be attributable to differences in participants, MRI scanner, magnetic field intensity and the data analysis methods between the two studies. The patients in our study were those with normal corrected visual acuity and large deviation angles of exotropia, without stereopsis, which can exclude the influence of confounding factors such as amblyopia. The magnetic field intensity in our study was 3.0 T, which has advantage over the 1T MRI intesity in Chan et al's study, because a high-field MR system provides higher signal/noise ratio, which results in increased spatial resolution and better imaging quality [22], [23]. Moreover, we used optimized VBM which is superior to the conventional VBM such as excluding the influence of non-cerebral tissues and correcting the volume changes during normalization [18].

There is no prior voxel-based analysis of DTI study in comitant strabismus, and the present study found that the FA value was decreased in right middle occipital gyrus and right supramarginal gyrus, indicating abnormalities of white matter fibers in these areas. White matter region with higher FA in strabismus patients was found in the right inferior frontal gyrus. The result was consistent with VBM in our study, which had changes primarily in right hemisphere white matter, especially occipital and parietal areas.

Previous animal experiments have demonstrated that the discordance of binocular visual stimulation caused by strabismus would influence the structure and function of visual cortex and nervous pathways during the critical period of visual plasticity. Studies with strabismus animal model found that the binocular neurons in V1 area were reduced in number in cat and monkey, while neurons activated by each eye were not affected [24], [25], [26], [27], [28], [29], [30]. Behaviorally, strabismus monkey manifest stereo-blindness [31]. Further investigations found that the binocular inhibitory site was in primary visual cortex, and this inhibition was mediated by GABA [32]. Moreover, evidence showed that strabismus caused extensive impairment of connections in the BA17 cortex during the critical period [33], [34], [35], as well as the impairment of BA18 horizontal connections and connections between BA17 and BA18, which in turn influenced stable establishment of neuron circuits in the visual cortex [9]. Short-term strabismus can also impact the development of visual cortex and fibrous connections. Trachtenberg et al. established a 2-day strabismus model in infant cat during peak visual development and found the reorganization of horizontal connections in the upper layer of the visual cortex [35]. Zhang et al. constructed 3-day strabismus in monkeys during the peak of the critical period and similarly demonstrated functional changes of V1 area, in which more neurons inhibited binocular stimulation [7]. Meanwhile, long range connections in advanced cortex were found to be more plastic, and these connections were prone to the exterior influences [7]. These investigations all suggest that strabismus can cause structural and functional abnormalities in the visual cortex, and impact the stable development of white matter tracts. Though our study showed that the structure and connections were impaired in the dorsal visual pathway in comitant strabismus patients, we can't provide further evidence for these changes were the cause or the result of aberrant information processing in comitant strabismus. In our study, the stereopsis of patients with comitant strabismus was severely impaired, and we think this is probably associated with the abnomalities of dorsal visual pathway.

Our results suggest that the cerebral impairment is mainly located in the right hemisphere, especially dorsal saptial processing pathway. Interestingly, studies have demonstrated that activated cerebral areas were primarily in right dorsal pathway when subjects were stimulated with stereogram. Fortin et al. found that the normally activated cerebral areas were right BA18, 19 and 7 (examined with positron emission tomography) while being stimulated with random dot stereogram [36]. Nishida et al. utilized functional MRI and found that the areas that were responsible for stereopsis were located in the dorsal region of right parietal-occipital lobule [37]. And Kwee et al. also found that the stereopsis areas were in the right parietal lobule and its surrounding areas with 3T functional imaging [38]. In our study, these stereopsis areas of the right hemisphere are likely to be impaired in these strabismus cases, leading to impaired stereopsis.

At present, the VBM and voxel-based analysis of DTI approaches are extensively used in neurological studies. However, there are some limitations in both methods: 1. With VBM, there is an image shifting phenomenon caused by inaccuracy of coregister, or insufficient or excessive smoothing. For instance, the analysis of gray matter difference found some masses in the white matter or cerebrospinal fluid regions [16]. 2. For voxel-based analysis of DTI, (1) the white matter diffusion is Gaussian in distribution in the DTI model, while the real white matter distribution in human brain is non-Gaussian in distribution [39], [40], [41]. (2) DTI describes each voxel (the size is usually 2.5×2.5×2.5 mm^3^) based on single diffusion tensor, which includes numerous components of axons and glial cells. Thus, the diffusion tensor is a mean value and DTI cannot distinguish crossed white matter fibers [17]. Therefore, with the improvement of VBM and DTI, more precise locations of cerebral impairment caused by strabismus can be identified.

In conclusion, our study explored the areas of cerebral impairment in adult patients with comitant extropia through VBM and voxel-based analysis of DTI methods, we found that the dorsal visual pathway was abnormal or impaired in patients with comitant exotropia.

## References

[1] Chew E, Remaley NA, Tamboli A, Zhao J, Podgor MJ (1994). Risk factors for esotropia and exotropia.. Arch Ophthalmol.

[2] Govindan M, Mohney BG, Diehl NN, Burke JP (2005). Incidence and types of childhood exotropia: a population-based study.. Ophthalmology.

[3] Robaei D, Rose KA, Kifley A, Cosstick M, Ip JM (2006). Factors associated with childhood strabismus: findings from a population-based.. Ophthalmology.

[4] Baker FH, Grigg P, von Noorden GK (1974). Effects of visual deprivation and strabismus on the response of neurones in the visual cortex of the monkey, including studies on the striate and parastriate cortex in the normal animal.. Brain Res.

[5] Crawford MLJ, von Noorden GK (1979). The effects of short-term experimental strabismus on the visual system in Macaca mulatta.. Invest Ophthalmol Vis Sci.

[6] Crawford ML, Smith EL, Harwerth RS, von Noorden GK (1984). Stereoblind monkeys have few binocular neurons.. Invest Ophthalmol Vis Sci.

[7] Zhang B, Bi H, Sakai E, Maruko I, Zheng J (2005). Rapid plasticity of binocular connections in developing monkey visual cortex (V1).. Proc Natl Acad Sci U S A.

[8] Kumagami T, Zhang B, Smith EL, Chino YM (2000). Effect of onset age of strabismus on the binocular responses of neurons in the monkey visual cortex.. Invest Ophthalmol Vis Sci.

[9] Mori T, Matsuura K, Zhang B, Smith EL, Chino YM (2002). Effects of the duration of early strabismus on the binocular responses of neurons in the monkey visual cortex (V1).. Invest Ophthalmol Vis Sci.

[10] Engelmann R, Crook JM, Löwel S (2002). Optical imaging of orientation and ocular dominance maps in area 17 of cats with convergent strabismus.. Vis Neurosci.

[11] Schmidt KF, Löwel S (2008). Strabismus modifies intrinsic and inter-areal connections in cat area 18.. Neuroscience.

[12] Chino YM, Cheng H, Smith EL, Garraghty PE, Roe AW (1994). Early discordant binocular vision disrupts signal transfer in the lateral geniculate nucleus.. Proc Natl Acad Sci U S A.

[13] Cheng H, Chino YM, Smith EL, Hamamoto J, Yoshida K (1995). Transfer characteristics of X LGN neurons in cats reared with early discordant binocular vision.. J Neurophysiol.

[14] Chan ST, Tang KW, Lam KC, Chan LK, Mendola JD (2004). Neuroanatomy of adult strabismus: a voxel-based morphometric analysis of magnetic resonance structural scans.. Neuroimage.

[15] McKee SP, Levi DM, Movshon JA (2003). The pattern of visual deficits in amblyopia.. J Vision.

[16] Busatto GF, Diniz BS, Zanetti MV (2008). Voxel-based morphometry in Alzheimer's disease.. Expert Rev Neurother.

[17] Assaf Y, Pasternak O (2008). Diffusion tensor imaging (DTI)-based white matter mapping in brain research: a review.. J Mol Neurosci.

[18] Good CD, Johnsrude IS, Ashburner J, Henson RN, Friston KJ (2001). A voxel-based morphometric study of ageing in 465 normal adult human brains.. NeuroImage.

[19] Shu N, Li J, Li K, Yu C, Jiang T (2009). Abnormal diffusion of cerebral white matter in early blindness.. Hum Brain Mapp.

[20] Merigan WH, Maunsell JH (1993). How parallel are the primate visual pathways?. Annu Rev Neurosci.

[21] Tootell RBH, Hadjikhani NK, Mendola JD, Marrett S, Dale AM (1998). From retinotopy to recognition: fMRI in human visual cortex.. Trends Cogn Sci.

[22] Scarabino T, Nemore F, Giannatempo GM, Bertolino A, Di Salle F (2003). 3.0 T magnetic resonance in neuroradiology.. Eur J Radiol.

[23] Alvarez-Linera J (2008). 3T MRI: advances in brain imaging.. Eur J Radiol.

[24] Hubel DH, Wiesel TN (1965). Binocular interaction in striate cortex of kittens reared with artificial squint.. J. Neurophysiol.

[25] Baker FH, Grigg P, von Noorden GK (1974). Effects of visual deprivation and strabismus on the response of neurones in the visual cortex of the monkey, including studies on the striate and parastriate cortex in the normal animal.. Brain Res.

[26] Blakemore C (1976). Modification of visual function by early visual experience.. Bull Schweiz Akad Med Wiss.

[27] Crawford MLJ, von Noorden GK (1979). The effects of short-term experimental strabismus on the visual system in Macaca mulatta.. Invest Ophthalmol Vis Sci.

[28] Singer W, von Grünau M, Rauschecker J (1980). Functional amblyopia in kittens with unilateral exotropia. I. Electrophysiological assessment.. Exp Brain Res.

[29] Crawford ML, Smith EL, Harwerth RS, von Noorden GK (1984). Stereoblind monkeys have few binocular neurons.. Invest Ophthalmol Vis Sci.

[30] Sengpiel F, Blakemore C, Kind PC, Harrad R (1994). Interocular suppression in the visual cortex of strabismic cats.. J. Neurosci.

[31] Crawford ML, von Noorden GK, Meharg LS, Rhodes JW, Harwerth RS (1983). Binocular neurons and binocular function in monkeys and children.. Invest Ophthalmol Vis Sci.

[32] Sengpiel F, Jirmann KU, Vorobyov V, Eysel UT (2006). Strabismic suppression is mediated by inhibitory interactions in the primary visual cortex.. Cereb Cortex.

[33] Löwel S, Singer W (1992). Selection of intrinsic horizontal connections in the visual cortex by correlated neuronal activity.. Science.

[34] Schmidt KE, Kim DS, Singer W, Bonhoeffer T, Löwel S (1997). Functional specificity of long-range intrinsic and interhemispheric connections in the visual cortex of strabismic cats.. J. Neurosci.

[35] Trachtenberg JT, Stryker MP (2001). Rapid anatomical plasticity of horizontal connections in the developing visual cortex.. J. Neurosci.

[36] Fortin A, Ptito A, Faubert J, Ptito M (2002). Cortical areas mediating stereopsis in the human brain: a PET study.. Neuroreport.

[37] Nishida Y, Hayashi O, Iwami T, Kimura M, Kani K (2001). Stereopsis-processing regions in the human parieto-occipital cortex.. Neuroreport.

[38] Kwee IL, Fujii Y, Matsuzawa H, Nakada T (1999). Perceptual processing of stereopsis in humans: high-field (3.0-tesla) functional MRI study.. Neurology.

[39] Beaulieu C (2002). The basis of anisotropic water diffusion in the nervous system - a technical review.. NMR Biomed.

[40] Assaf Y, Basser PJ (2005). Composite hindered and restricted model of diffusion (CHARMED) MR imaging of the human brain.. Neuroimage.

[41] Jensen JH, Helpern JA, Ramani A, Lu H, Kaczynski K (2005). Diffusional kurtosis imaging: the quantification of non-gaussian water diffusion by means of magnetic resonance imaging.. Magn Reson Med.

